# A behavioural change intervention study for the prevention of childhood obesity in South Africa: protocol for a randomized controlled trial

**DOI:** 10.1186/s12889-020-8272-1

**Published:** 2020-02-04

**Authors:** Natisha Dukhi, Benn Sartorius, Myra Taylor

**Affiliations:** 1 Social Aspects of Public Health, Human Sciences Research Council, Cape Town, South Africa; 2 Discipline of Public Health Medicine, School of Nursing and Public Health, University of KwaZulu-Natal, Durban, South Africa; 3 Faculty of Tropical and Infectious Diseases, London School of Hygiene and Tropical Medicine, London, UK

**Keywords:** Children, Adolescents, School, Diet, Nutrition, Physical activity, Overweight, Obesity, Cluster randomized controlled trial

## Abstract

**Background:**

South Africa is currently undergoing a nutrition transition, and overweight and obesity is on the increase in South African children. Urbanization and other health determinants have led to reduced physical activity and unhealthy eating that have increased the risk of adverse chronic health conditions. This study aims to provide evidence of the effectiveness of a school-based intervention study that targets diet and physical activity for the prevention of child and adolescent overweight and/or obesity.

**Methods:**

We will employ a mixed method study design which is divided into two phases. Phase 1, namely the qualitative elicitation research phase will inform the development of the quantitative intervention phase (phase 2), consisting of a cluster-randomized trial, based on input from key stakeholders. The study will be undertaken in 16 government-funded primary schools in the iLembe district of KwaZulu-Natal, South Africa. The study will target learners in Grades 4 and 7, their parents, Life Orientation educators, school principals and members of school governing bodies. Assessment for the primary objective (BMI Z scores), and the secondary objectives (change in knowledge, attitudes and behaviours regarding diet and physical activity) in both study arms will be conducted at baseline in March 2020 and at the end of the study in October 2020.

**Discussion:**

The study will be a novel combined mixed methods/RCT design that focuses on diet, physical activity school and family-based interventions in the context of rapidly increasing overweight and obesity prevalence in KwaZulu-Natal. To encourage behaviour change and management of malnutrition, education including diet and physical activity, is an important strategy that must be considered. Nutrition education extends beyond the dissemination of food information; it includes addressing the needs of participants, empowers and encourages decision-making and choice of foods, change in nutrition attitudes, beliefs and influences based on resources available and within cultural boundaries.

**Trial registration:**

Pan African Clinical Trial Registry PACTR201711002699153. Protocol registered on 16 November 2017.

## Background

Contributing to the over nutrition epidemic, an estimated 340 million children and adolescents aged 5–19 years were overweight or obese in 2016 [1]. The World Health Organization (WHO) global nutrition targets for 2025, especially target 4, has a specific focus on preventing an increase in childhood overweight [2]. The rapid increase in overweight and obesity in children and adolescents, largely due to modifiable risk factors such as unhealthy eating and physical inactivity, is strongly influenced by the physical environment, as well as cultural, social and economic factors [3]. It is estimated that by the year 2020, lifestyle-related non-communicable diseases (NCDs) will account for 60% of the burden of disease, and 70% of deaths worldwide, if adequate health promotion intervention programmes are not established. To reduce this chronic disease burden, modification of one’s lifestyle, promotion of prevention measures against NCDs and improved dietary habits and physical activity are important [4, 5]. Furthermore, a cause for public health concern is the increasing trend of child and adolescent weight gain as they transition into adulthood. Adolescents, 10–19 years of age, as defined by the WHO, are targeted for interventions as it is during this growth and development period that health attitudes, beliefs and behaviours are established and continue into adulthood [3]. The adolescent years have been referred to as the “tipping years” by Hanvey et al., [6], where co-morbidities such as high blood pressure, diabetes, stroke, cardiovascular disease and cancer may appear as short term health consequences [7].

In an effort to reverse and stop the increased trend of overweight and obesity, children should be brought up in a health promoting environment where instead of traditionally following the choices of others they can make informed choices about their health [3]. The school is viewed as an ideal health-promoting environment that can develop children and adolescents physically, emotionally and socially [8]. Literature on school obesity interventions exists, and while some literature that specifically focuses on diet and physical activity exists, such tend to focus on anthropometric measures such as body mass index (BMI) only, self-efficacy and some positive evaluations and outcomes [9–11]. In South Africa, there have been minimal obesity intervention studies, with some focusing on physical activity only, and nutrition interventions having either a food questionnaire to identify food choices or BMI measurements only. The HealthKick study, comprising of diet and physical activity interventions is the only known study in South Africa that aimed to address the prevention of overweight in children and reduce the risk of chronic diseases such as type 2 diabetes. This study identified significant improvement in self-efficacy and nutrition knowledge but not in children’s nutrition behaviour. Limitations of the study included lack of parent participation, as well as the authors suggesting that creating a healthier food environment within and around the school was important [12]. The study, in addition to other international studies, has been helpful in assisting in the development of the iLembe School Physical Activity and Nutrition (i-SPAN) study. The i-SPAN study identified the strength and weaknesses of South African studies and while these studies were implemented with some success in low-income areas, literature for school-based diet and physical activity interventions is still limited in the African setting [13–15].

Therefore, there is a need to develop and implement interventions that are specific for lifestyle diseases and conditions that target the child groups relevant to South Africa. Such interventions may be difficult to conduct and sustain due to a lack of funding and resources. Thus, the i-SPAN study has been developed and will be conducted in the poverty-challenged iLembe District in the province of KwaZulu-Natal in South Africa. The elicitation research phase will inform the development of an intervention phase that will design, implement and assess the effectiveness of school-based interventions that focus on diet and physical activity in children and adolescents in Grades 4 and 7 (aged 9–15 years). It is during these years that most girls (8–13 years) and boys (10–15 years) enter puberty, reaching a growth peak that results in a healthy BMI and weight [16].

### Trial aims and objectives

The aim of this cluster randomized controlled trial (RCT) is to provide evidence of the effectiveness of diet and physical activity interventions conducted in schools in preventing overweight and obesity in children and adolescents.

### Specific objectives

#### Phase one: elicitation research

1. To assess the health promotion status of children and adolescents in the iLembe district schools (what children know about diet and exercise, and their diet and physical activities), explore and understand the school children’s attitudes, cultural beliefs, vision and ideas regarding obesity, and identify the extent to which health promotion has been utilized in the schools regarding diet and physical activity.
2. To identify facilitating and constraining factors for health promotion (diet and physical activity) in the schools in iLembe district according to the key stakeholders such as Department of Basic Education (DoBE), Department of Health (DoH), Department of Sports and Recreation and other stakeholders such as the Health Promotion Coordinators, District Nutrition Coordinator, School Health Teams.
3. To design and implement diet and physical activity school-based interventions to reduce obesity in school children.

#### Phase two: intervention

4. To assess the effectiveness of the i-SPAN study in children and adolescents aged 9–15 years by comparing the intervention and control arms at baseline and at the end of the study regarding:
  - BMI Z scores.
  - Proportion of children and adolescents classified as overweight and obese.
  - Knowledge, attitude and behaviours regarding diet and physical activity.
5. To describe and evaluate the outcomes of the interventions for scientific dissemination.

## Methods

The protocol has been developed using the Standard Protocol Items: Recommendations for Interventional Trials (SPIRIT) guidelines [17] (Additional file 1).

### Design

**Fig. 1:**
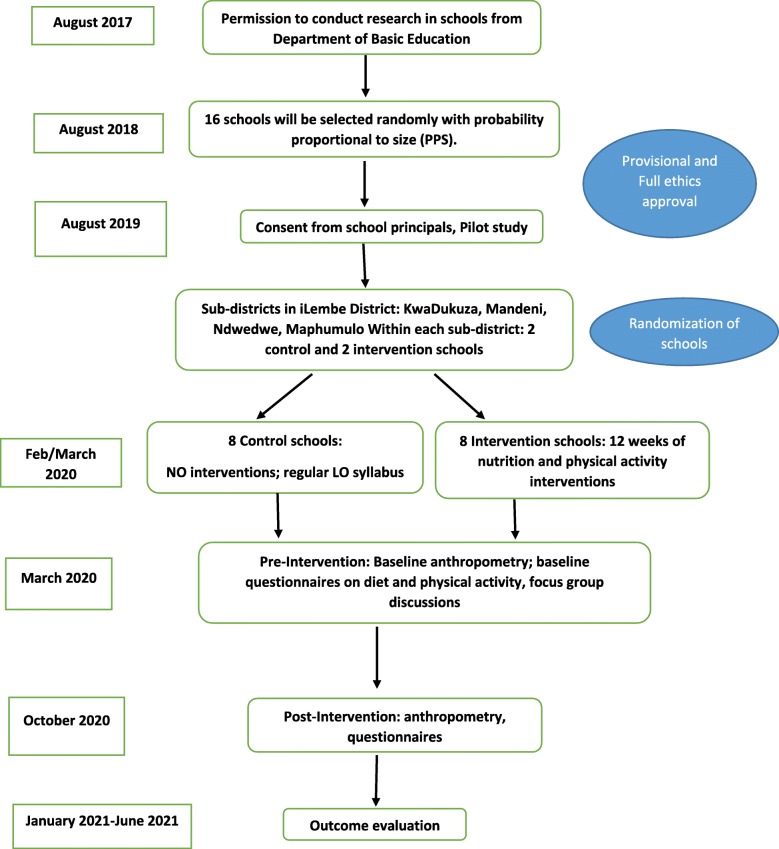
Study design of the i-SPAN study

The study is a cluster randomized controlled trial, with both quantitative and qualitative components, to determine the effectiveness of the i-SPAN study to prevent overweight and/or obesity in children and adolescents (Fig. 1).

### Participants and recruitment process

The participants of the study will include children in grade four (aged 9-11 years) and grade seven (aged 12-15 years), as well as parents/primary caregivers, Life Orientation (LO) educators, principals, members of the community, the latter through the school governing body (SGB). When the data has been collected meetings will be held with the SGBs (from whom permission will be required prior to the study) to inform them of the results and get their input on the way forward. They will also be the link with the wider community. Other stakeholders will include DoBE, DoH, Sports and Recreation, Health Promotion Coordinators, the District Nutrition Coordinator and School Health Teams in iLembe district, KwaZulu-Natal. The study setting will be primary schools in iLembe district.

A complete list of all schools with grade four and seven children in iLembe district will be provided by the Provincial Department of Basic Education. The list includes over 400 primary schools from which 16 schools will be selected randomly with probability proportional to size (PPS). There will be four schools from each of the four sub-districts, which will be randomly assigned into the two study arms (control *n* = 8, intervention n = 8). Two schools will receive the intervention and two schools will serve as controls in each of the four sub-districts. All children and adolescents in grade four and seven attending these schools and their parents/primary caregiver will be invited to participate in the study. Children and adolescents must provide assent in writing via the consent forms. Parents/caregiver must also provide consent for participation in the study for the children/adolescents and themselves (Additional file 2). South African schools are arranged into quintiles determined by the National Treasury, based on scores reflective of the poverty levels where a community is situated. The DoBE uses these poverty classifications that range from quintiles 1–5, where quintile five is the least poverty-stricken and one is the most poverty-challenged quintile, to classify schools. Most of the primary schools in iLembe district are from quintiles 1 and 2, hence it is important to understand the daily challenges these communities face regarding housing, socio-economic status, employment, as well as health outcomes as these are regarded as schools with low socio-economic status and exempt from school fees [18].

### Sample size/power calculation

The school is the unit of the study. The power/sample size calculation was performed using a test for two means in a cluster randomized design function implemented in PASS 12 [19]. A power calculation indicated that with 8 schools in each arm and 68 participants per cluster, the study would have 90% power (1-β) to detect an underlying medium standardized difference between the means (effect size) of 0.3 following the intervention, at a two-sided 5% significance level (α), assuming an intra-class correlation coefficient (ICC) of 0.05 or 5% [20, 21].

### Randomization

The school is the unit of the study. A biostatistician who does not have direct contact with study participants will generate the random allocation sequences using a computerized random number generator. After the inclusion of each cluster, the allocation of that particular cluster will be provided to the study coordinator. There will be no crossover between study arms.

### Inclusion/exclusion criteria

The inclusion criteria include: a) Children and adolescents in grades four and seven (aged 9–15 years). b) Parent/primary caregiver of the participating children/adolescents. c) Children and adolescents with physical disabilities will be included in the study but excluded from the anthropometry.

### Study components and implementation

The study will consist of two phases: Phase 1 is a baseline elicitation phase using a qualitative paradigm, whereby the formative assessment will inform the development of the intervention for use in phase 2. A thorough search and review of available scientific literature will be carried out to determine what is currently being done regarding learners, particularly in respect to health promotion concerning physical activity and dietary habits of school-going learners in South Africa and specifically in iLembe district, KwaZulu-Natal. During the elicitation phase focus group discussions (FGDs) will be conducted with selected members of the SGB, as well as selected children and adolescents in grades four and seven to explore current practices at each school. In-depth interviews will be conducted with principals regarding nutrition and PA at school. Based on the information obtained from the elicitation research phase, a plan for the intervention phase based on the Social Cognitive Theory (SCT) framework [22] will be developed and implemented. During the intervention phase structured questionnaires will be given to the children and adolescents in grades four and seven, the LO educators and the parents/primary caregivers of the participating children and adolescents to determine their understanding, knowledge, attitudes and behaviours regarding diet and physical activity at baseline and at the end of the study.

### Training

Fieldworkers will be recruited for the study and training will continue in 2020. A training manual will be compiled by the principal investigator and co-investigators of the study, who will also facilitate the training. During the training fieldworkers will be given an in-depth background about the significance of the study and the scope of malnutrition in the South African and the global context. They will be carefully trained on the study instruments that will be used such as FGDs and structured interview questions, administering of the intervention questionnaires and the measuring of height and weight in children and adolescents. Fieldworkers must be fluent in isiZulu and English. They will also be trained on their role in the study, as well as their interactions with members of the school, community and stakeholders such traditional leaders. Once the aims of the study and the fieldworker role is understood they will be given the information and consent forms in both English and isiZulu to role play as these have been selected as the two mediums of communication in the schools. During role play various scenarios will be created to allow fieldworkers to be adequately prepared in their approach to the schools in the study. Further training will be conducted with fieldworkers whereby they are introduced to the equipment that will be used for the study. Repeated training and a pilot phase will focus on achieving inter- and intra-rater reliability of measures.

### Pilot study

Prior to the study, the survey instruments will be piloted in one school that will not be included in the study by the researcher/fieldworkers. It will be important to test the questionnaire and measurement instruments to eliminate any ambiguity in the questions asked in the questionnaire and to correct any difficulties experienced before the main study. The pilot study will follow the same procedures as the main study but use different schools. The data from the pilot study will not be included in the main study. Confidentiality of the participants will be maintained at all times throughout the pilot and main study. The participant’s name or any personal details relating to the participants will not be mentioned in any publication, report or meetings where results of the researcher are being presented.

### Data collection instruments and techniques

Table 1 below identifies and describes the measures to be assessed and techniques utilized in the i-SPAN study.

**Table 1:** Measures to be assessed and techniques utilized in the i-SPAN study

Measures assessed	Target audience	Phase of study	Instrument or standard practice utilized and its description
Focus group discussions (FGDs)	a. Selected school governing body members b. Selected learners in grades four and seven	Phase 1- Elicitation phase	An interview schedule has been developed by the research team and will be used as a guide during the FGDs to explore current practices at each school. All FGDs will be audio-recorded and conducted in the language of choice by fieldworkers. The audio-recordings will be sent to an external transcriber who will transcribe verbatim.
Structured in-depth interviews (IDIs)	School principals	Phase 1- Elicitation phase	An interview schedule has been developed by the research team and will be used as a guide during the IDI regarding health promotion in schools.
Questionnaire	a. LO educator b. Learners c. Parents of learners	Phase 2- Intervention phase	The questionnaires will be designed and adapted from questionnaires used in previous school intervention studies [23–25]. a. Educators will complete a pre and post intervention questionnaire based on diet and physical activity. b. Demographic data, diet and physical activity questionnaires for children and adolescents will be collected. Questionnaires will include pictures for food choices to enhance the learning activities. c. Same as b. Questionnaires will be sent home with the participating learners and asked to be returned within the same school week Reliability of the questionnaire will be ensured as follows: During the training of the fieldworkers’ changes/amendments to the questions will be made to ensure that there is clarity and no ambiguity in the questions being asked of all participants. The questionnaires will be translated into isiZulu by a person familiar with the content and back-translated into English by an independent translator. The questionnaire will be conducted in the language of choice (English or isiZulu) of the participant. The activities during the intervention will be conducted in English but as the children are young, the fieldworkers will be able to assist them in isiZulu whenever needed. Questions will further be refined during and after the pilot study to further detect any ambiguity or difficulty.
Anthropometry	Weight and height for all participating children and adolescents	Phase 2- Intervention phase	Weight and height will be measured using a digital weighing scale, and stadiometers. (Model no. 210/217/876; Seca, Hamburg, Germany). The weight of the participant will be taken to the nearest 0.1 kg for accuracy. The participants will be weighed with minimal clothing, and preferably with an empty bladder and before a meal [26]. Measurements using the stadiometer will be taken to the nearest 0.1 cm for accuracy. The participants will be measured without shoes. Participants are required to stand with their legs straight, heels and the height measure touching at the back, arms at the sides, relaxed shoulders, with chin level to ground and looking straight ahead [26]. Weight and height measurements will be taken in duplicate for accuracy and a correct average. The body mass index (BMI) for age Z scores, according to the 2006 WHO Growth Standards for children, will be used to classify being at risk of overweight with a Z score of SD 1, overweight with a Z score SD > 1 and obese as a Z score SD > 2 [27].
Workshop	LO educators	Phase 2- Intervention phase	To facilitate study implementation, educators will be invited to attend a two-day training workshop at the university to assist educators in understanding and delivering the classroom lessons as part of the intervention. They will also be provided with the findings of the baseline data to assist them.
Health Promotion Toolkit	Schools randomized into intervention arm	Phase 2- Intervention phase	The toolkit consists of the learner pamphlet, the educator manual, and sports box. a. The pamphlet includes all relevant information for learners and simplified regarding overweight and obesity. b. Instead of being a stand-alone school subject, physical education became one of the four learning outcomes of the LO subject of the new curriculum, thus not receiving adequate attention as an important part of healthy living for the learner outcomes [28]. The educator manual has been developed and adapted from teaching manuals in previous nutrition and school-based intervention studies [29, 30]. The manual consists of collated materials that constitutes the learner outcomes of the Life Orientation curriculum. The LO learner outcomes, as per the South African National Curriculum, include health promotion, as well as social, personal and physical development and movement [28]. The manual explains the significance of the obesity epidemic in South Africa and in children, the purpose of the manual, the goals and objectives of the i-SPAN study, as well as the monitoring and evaluation processes. The intervention and lesson plans are designed so that educators have some level of flexibility as topics may overlap. Activities are also included after every lesson to assist educators and make the lessons more enjoyable for the learners. If lessons similar to those in the manual are being conducted, then the manual will serve as a reinforcing tool for the educators. However, if the nutrition and physical activity components are not part of a school LO classroom lesson plan, then the educators will be introduced to and assisted with understanding the importance of the healthy lifestyle components during the educator workshops that will take place prior to commencement of the study. c. The Department of Sport and Recreation South Africa actively promotes the options of indigenous games as recreational or formal games. There are ten games have been identified as part of an indigenous games national project. These include: dibeke (a running ball game); diketo (a coordination game); kgati (a rope jumping game); ncuva (a board game); morabaraba (a board game); jukskei (a throwing and target game); kho-kho (a running game); Iintonga (a stick fighting game); arigogo (similar to rounders) and drie stokkies (running and jumping game). It is important to capitalize on such opportunities as they serve as inexpensive, culturally and context-specific tools that contribute to child health. Therefore, members from the Department of Sports and Recreation will assist the educators and fieldworkers during the physical activity times (this will be negotiated with LO teachers and principals for suitable times such as during breaks, after school and during the specific physical activity times as indicated by stakeholders within the four sub-districts). It will not be possible to include all games but the Department of Sports and Recreation has proposed teaching the learners at least a few of the indigenous games that they are familiar with, their parents know of or basically introducing it as a novel experience to them.

All instruments and measurements will be conducted for each school in a scheduled visit, unless alternate arrangements are made to revisit the school for data collection. The control schools will not receive the health promotion toolkit but will follow the regular LO syllabus

### Behavioural outcomes

We believe there are two important behaviour change outcomes that can be achieved from the study, i.e. healthy food choices and increased food variations, and increased physical activity participation during school hours. Table 2 below describes the two behavioural outcomes and the anticipated change based on the SCT model [22].

**Table 2:** Social cognitive theory constructs embedded in the interventions

Use of Social Cognitive Theory grounded in the interventions			
SCT constructs	Cognitive component	Behaviour component	Environment component
Knowledge of health benefits and risks [22, 31, 32]	Assess the health promotion status in the iLembe district schools and identify the extent to which health promotion has been utilized in the schools (regarding diet and physical activity) by interviewing principals and conducting an interview with principals at baseline. Nutrition education based on current knowledge- questionnaires that identifies health benefits and risks will be conducted at baseline and post-intervention to assess if there is a significant change in knowledge based on the interventions.	Healthy eating activities; physical activities Food evaluation: classroom lessons will be designed to assist learners on the risks associated with unhealthy eating, sedentary behaviours, and encourage the learners to bring healthy snacks to school. As the children and adolescents may receive either breakfast or lunch from the school feeding scheme, they can evaluate their meals accordingly.	Food policy to be reviewed or implemented; less energy-dense foods and sugar-sweetened drinks to be sold in school tuck shops and by vendors.
Perceived self-efficacy [22, 33]	Promote positive body image through classroom lessons, hand puppets; children can become empowered to make correct decisions regarding their health. Through having the knowledge of healthy eating options, unhealthy foods and health risks, as well as sedentary behaviour linked to health conditions, children become more aware of choice and the intention to making correct choices and changing behaviours/attitudes that benefit them e.g. less intake of sweetened juices, energy-dense snacks when given the options	Find differences between foods that are nutrient dense and foods that are low in nutrients and energy- dense- use colouring in competitions to colour in healthy options, role-play of good and bad eating and lifestyle habits, sporting activities that include netball and soccer tournaments and prize-giving.	Interactive homework to work with parents, Barriers to change questionnaire
Outcome expectations [22, 33]	Classroom lessons that focus on fruit and vegetable intake, the Food Pyramid, and how to achieve a balance through healthy eating & physical activity	Taste-testing activities, preparing simple fruit and vegetable snacks, aerobics activities, Self-evaluation of current fruit and vegetable intake	Motivational group discussions will also be conducted with both learners and educators Educators serve as school champions and role models Observing parents cooking at home. Use local media to create health awareness regarding healthy eating and activities
Perceived facilitators [22, 34]	Nutrition education based on current knowledge and experiences, discuss ways to achieve balance through healthy eating & physical activity	Provide opportunities for learners to choose healthier food and snack alternatives (taste-testing)	Exposing learners to healthier foods, participation in learning activities with peer groups, parents and learners to create after school supper menus modified to be lower in fat & higher in fruits and vegetables, choosing water or juice offered instead of cool drink, taking into consideration the costs. Work with vendors to identify unhealthy foods and drinks and replace them with fruits, healthy snacks, encourage learners to drink more clean water

### Mediating and moderating variables

The i-SPAN learner questionnaire will be used to assess any potential mediators, as part of the process evaluation, to identify any possible processes that may mediate diet and physical activity changes. The questionnaire consists of different sections that are able to assess knowledge, attitudes and beliefs, as well as the mediating behaviours of the child and parent that may affect diet and physical activity levels. Possible moderating variables such as sex, weight status and healthy lifestyle behaviours will be obtained from the baseline data of the study. The questionnaires will be administered during the classroom LO lessons by the fieldworkers to ensure that the learners understand the questions.

### Process evaluation

This phase will seek to identify the acceptability and impact of the interventions on the learners, educators and parents. It will also explore the extent to which the health promotion toolkit will be utilised in the intervention schools. Various factors such as facilitating and constraining factors to implement toolkit at the schools, the implementation process, times and durations of implementation will be taken into account. Therefore, to conduct the process evaluation a mixed methods approach will be utilised.

### Planned data collection

The data will be de-identified and available for secondary analyses for student projects. Electronic data quality will be ensured by checking completeness of required variables throughout the data collection process, and will comprise: number of participants correspond to number of questionnaires completed. Absenteeism will be recorded. For data analysis and results, all personal identifiers of participants will be removed during reporting phases. All data will be handled and coded only by the principal investigator and co-investigators who will serve as a data monitoring committee for the study. The principal investigator will also be responsible for auditing trial conduct, as well as any adverse events during the trial. Reports will be presented and made available to the various stakeholders mentioned previously and UKZN to assist in managing and preventing overweight and obesity in childhood. The data will also serve to assist the community i.e. iLembe district in managing and preventing overweight and obesity in children and adolescents. Findings of the study will also be disseminated through conferences and peer-reviewed publications. During the survey if learners are identified as overweight or obese, will be referred for appropriate care to local health facilities as per Department of Health levels of care.

### Planned statistical analysis

#### Descriptive and analytical statistics

The use of weight and height, BMI calculations, completed questionnaires, arising from the interventions will use both descriptive and inferential statistics to analyse the data. To ensure succession of cluster randomization, the presence of any significant differences between the control and intervention cluster for the baseline characteristics of the participants such as sex, age, etc., will be examined using bivariate mixed effect regressions to account sufficiently for the clustered design. Data will be processed and analysed using Stata 13.0 SE. Mixed effects using generalized linear and logistic modelling approaches will be used to compare continuous and binary endpoints respectively among students in schools in intervention and control arms of the study. A within school random effect will be incorporated to account for correlated (repeated) measurements within each school to correctly estimate the significance when comparing pre- and post-intervention periods. Outcome variables include change in BMI at baseline and end of study, dietary intake, time spent in sedentary behaviours, diet and physical activity self-perception and practices, including overall knowledge and attitude regarding diet and physical activity- significant levels and stratified analyses.

#### Qualitative analysis

All transcribed data from the FGDs and in-depth interviews will be managed using NVIVO8. The data will be imported to the NVIVO8 program, linked to memos where coding will occur. Topics and concepts will be coded i.e. to capture information about happenings, ideas or specific topics, and nodes will be created to mark relevant information to be analysed or searched [35]. Thereafter, data will be manually analysed for emerging common themes.

## Discussion

To our knowledge, this is the only other RCT to be conducted in South Africa, and the first in KwaZulu-Natal, that includes diet and physical activity school-based interventions. To encourage behaviour change and management of malnutrition nutrition, education (that includes diet and physical activity) is an important strategy that must be considered [36]. Nutrition education extends beyond the dissemination of food information; it includes addressing the needs of children and adolescents, parents and the community, empowers and encourages decision-making and choice of foods, encourages change in attitudes, beliefs and influences based on resources available and the needs of individuals, as well as improving and promoting the nutrition knowledge within cultural boundaries [37, 38]. Nutrition education serves to not only impart important nutrition information and health messages but rather involves the participants to engage in activities and/or interventions that will improve nutrition [39]. If the i-SPAN study is effective, this RCT will be implemented to other schools, including the study control schools in iLembe district and outside of iLembe as part of the prevention and management of childhood obesity in KwaZulu-Natal.

## Supplementary information

**Additional file 1.** The Standard Protocol Items: Recommendations for Interventional Trials (SPIRIT) guidelines

**Additional file 2.** Information sheet and participant consent

## Data Availability

Not applicable.

## Abbreviations

BMI: Body mass index
BREC: Biomedical Research Ethics Committee
DoBE: Department of Basic Education
DoH: Department of Health
FGD: Focus group discussion
i-SPAN: iLembe School Physical Activity and Nutrition
LO: Life Orientation
NCDs: Non-communicable diseases
PPS: Probability proportional to size
RCT: randomized controlled trial
SCT: Social Cognitive Theory
SPIRIT: Standard Protocol Items: Recommendations for Interventional Trials
UKZN: University of KwaZulu-Natal
WHO: World Health Organization
