# Supplementary material for: A behavioural change intervention study for the prevention of childhood obesity in South Africa: protocol for a randomized controlled trial
Source: BMC Public Health. 2020 Feb 4;20:179. doi: 10.1186/s12889-020-8272-1 (PMC7001200; doi:10.1186/s12889-020-8272-1)
Supplement: Supplementary file 2 — Additional file 2. Information sheet and participant consent [file 12889_2020_8272_MOESM2_ESM.doc]

## INFORMATION SHEET AND CONSENT TO PARTICIPATE IN RESEARCH STUDY

## INFORMATION SHEET FOR PARENT/PRIMARY CAREGIVER

Title of study: **The iLembe School Physical Activity and Nutrition (i-SPAN) study for the prevention of childhood obesity.**

Good day/afternoon

My name is Prof Myra Taylor from the Discipline of Public Health Medicine at the University of KwaZulu-Natal (UKZN), telephone: 0312604499, [taylormyra@gmail.com](mailto:taylormyra@gmail.com)

You are being invited to participate in a study that involves research about the health of children and specifically obesity. We hope that this will tell us more about reducing obesity in school children and what improvements can be made in future.

You are being asked to consider taking part in a research study that will measure the growth of children aged 9-15 years, and help children to make good food choices and enjoy some exercise. From recent studies, we know that unhealthy (junk) foods and no exercising can make children become too fat which cause health problems. If children continue to eat unhealthily and do not do some exercise, then they can become obese adults and have a lot of problems such as hypertension and diabetes. By speaking to you and the children participating in activities at school that will teach them to eat healthy foods, play sport, we hope to see a good change in the health of the children and help them become healthy adults.

Our study will take place over 3 years and we will also be working with the school principals, teachers and members of the school governing body at the schools in iLembe district. We hope to work with at least 1100 children aged 9-15 years to help us find out if children in this age group are overweight or obese. From the 16 schools chosen for the study, your child may be in the control or intervention school group. Below is a description of the control and intervention schools.

Control schools: The learners will complete a questionnaire at the beginning and end of the study. On completion of the questionnaire they will receive a fruit juice. We will also take their height and weight measurements at the beginning and the end of the study.

Intervention schools: The learners will complete a questionnaire at the beginning and end of the study. On completion of the questionnaire they will receive a fruit juice. We will also take their height and weight measurements at the beginning and the end of the study. We will randomly select some learners to participate in a focus group discussion and they will be recorded using an audio-recorded. The learners will also participate in the intervention which will take place over 12 weeks. The interventions will involve games, class room lessons, competitions and other fun activities to help the children make good health choices. Even if your child is not obese, we will help all the children taking part in the study to eat correctly and to play simple sport during school hours. We will also work with your child to not only be healthy and active at school but to also help your child for their own health at home. The study will be funded by the National Research Foundation (NRF) and all things bought for the school activities will remain at the schools for the children to enjoy and stay healthy. Parents will be sent a questionnaire on diet and physical activity with the child to complete at home and return to the school.

***Risks & Benefits***

There is potential risk in being in this study as your child may feel uncomfortable during discussions on obesity if they are chubby. We do not want them to feel ashamed or stigmatized in any way. If the child feels uncomfortable or embarrassed, we will help and motivate them to better their health.

We will also work with the school health teams that are trained to appropriately counsel the children if they feel stigmatized and to offer referral to health facilities where necessary.

We hope that the study will create the following direct benefits: for children to eat less or no junk foods and eat healthily, and to watch less television and play more sport and exercise at school and at home. Scientific benefits of the study include the reduction of obesity in children in iLembe district. If we find health problems through our measurements and questions we will, with your permission, refer your child to your nearest clinic. You are free not to answer any questions that may distress you.

***Voluntary participation***

You are free to choose for you and your child/children to be part of the study. If you do not want to be part of the study, that is ok. If you want to stop at any time, that is ok. There will be no harm to you or your family if you stop. There is no payment for being part of this survey.

***Privacy and disclosure of information***

The answers we collect will be kept private. The names of anyone taking part or any personal details will not be shared with others or mentioned in any reports or meetings where results of the survey are being shared. We will ask your permission before we refer you to community workers or the clinic. Informed written consent will be obtained from primary caregivers, and confidentiality maintained. Participants may choose not to answer certain questions. Field workers will be trained to maintain confidentiality.

This study has been ethically reviewed and approved by the UKZN Biomedical Research Ethics Committee (approval number BCF555/17).

In the event of any problems or concerns/questions you may contact the researcher Prof Myra Taylor at 0312604499 or email at [taylormyra@gmail.com](mailto:taylormyra@gmail.com) or the UKZN Biomedical Research Ethics Committee, contact details as follows:

BIOMEDICAL RESEARCH ETHICS ADMINISTRATION

Research Office, Westville Campus

Govan Mbeki Building

University of KwaZulu-Natal

Private Bag X 54001, Durban, 4000

KwaZulu-Natal, SOUTH AFRICA

Tel: 27 31 2602486 - Fax: 27 31 2604609

Email: [BREC@ukzn.ac.za](mailto:BREC@ukzn.ac.za)

------------------------------------------------------------------------------------------------------------------

**CONSENT FOR PARTICIPATION IN RESEARCH**

**CONSENT FORM FOR PARENT/PRIMARY CAREGIVER**

I, _______________________________ (parent/primary caregiver name) have been informed about the study entitled the iLembe School Physical Activity and Nutrition (i-SPAN) study for the prevention of childhood obesity.

I understand the purpose and procedures of the study.

I have been given an opportunity to answer questions about the study and have had answers to my satisfaction.

I declare that my and my child’s participation in this study is entirely voluntary and that I may withdraw at any time without affecting any treatment or care that I would usually be entitled to.

I have been informed about any available compensation or medical treatment if injury occurs to me as a result of study-related procedures.

If I have any further questions/concerns or queries related to the study, I understand that I may contact the researcher at Prof Myra Taylor at 0312604499 or email at [taylormyra@gmail.com](mailto:taylormyra@gmail.com)

If I have any questions or concerns about my rights as a study participant, or if I am concerned about an aspect of the study or the researchers then I may contact:

**BIOMEDICAL RESEARCH ETHICS ADMINISTRATION**

**Research Office, Westville Campus**

**Govan Mbeki Building**

**University of KwaZulu-Natal**

**Private Bag X 54001, Durban, 4000**

**KwaZulu-Natal, SOUTH AFRICA**

**Tel: 27 31 2602486 - Fax: 27 31 2604609**

**Email:** [**BREC@ukzn.ac.za**](mailto:BREC@ukzn.ac.za)

**____________________ ____________________ ____________________**

**Name of Interviewer Signature of Interviewer Date**

**____________________ _____________________ ____________________**

**Name of Participant Signature of Participant Date**
